# General anesthesia with local infiltration reduces urine retention rate and prolongs analgesic effect than spinal anesthesia for hemorrhoidectomy

**DOI:** 10.3389/fsurg.2024.1288023

**Published:** 2024-01-19

**Authors:** Chun-Yu Lin, Yi-Chun Liu, Jun-Peng Chen, Pei-Hsuan Hsu, Szu-Ling Chang

**Affiliations:** ^1^ Division of Colorectal Surgery, Department of Surgery, Taichung Veterans General Hospital, Taichung, Taiwan; ^2^ School of Medicine, National Defense Medical University, Taipei, Taiwan; ^3^ Institute of Clinical Medicine, National Yang Ming Chiao Tung University, Taipei, Taiwan; ^4^ Department of Radiation Oncology, Taichung Veterans General Hospital, Taichung, Taiwan; ^5^ Department of Post-Baccalaureate Medicine, College of Medicine, National Chung Hsing University, Taichung, Taiwan; ^6^ Biostatistics Task Force of Taichung Veterans General Hospital, Taichung, Taiwan; ^7^ Department of Medical Research, Taichung Veterans General Hospital, Taichung, Taiwan; ^8^ Department of Anesthesiology, Taichung Veterans General Hospital, Taichung, Taiwan; ^9^ Institute of Emergency and Critical Care Medicine, National Yang Ming Chiao Tung University School of Medicine, Taipei, Taiwan

**Keywords:** hemorrhoidectomy, anorectal surgery, general anesthesia, local infiltration, urine retention, pain score

## Abstract

**Introduction:**

Postoperative pain and complications pose significant challenges following a hemorrhoidectomy. Attaining effective anesthesia with minimal complications is crucial. The ideal anesthesia method for ambulatory hemorrhoidectomy remains uncertain. This study aimed to investigate whether the combination of general anesthesia plus local infiltration (GAL) is associated with lower complications and reduced pain compared to spinal anesthesia (SA) in the context of hemorrhoidectomy.

**Methods:**

This retrospective single-center cohort study, conducted in a tertiary medical center in East Asia, evaluated excisional hemorrhoidectomies performed between January 1, 2017, and March 31, 2023, utilizing GAL or SA. Data on the six most common complications-pain, constipation, acute urine retention (AUR), bleeding, nausea, and headache-were extracted from medical records. A total of 550 hemorrhoidectomies were included: 220 in the GAL group and 330 in the SA group. Patient characteristics were comparable between the two groups.

**Results:**

The AUR rate was significantly lower in the GAL group compared to the SA group (15.5% vs. 32.1%, *P* < 0.001). Although the proportion of pain scores ≥4 did not differ significantly between the GAL and SA groups (36.2% vs. 39.8%, *P* = 0.429), the pain score curve indicated a stable trend. Overall, the GAL group exhibited a lower rate of adverse effects (56.9% vs. 67.4%, *P* = 0.023). There were no significant differences in the rates of other complications and emergency department readmission between the GAL and SA groups.

**Discussion:**

GAL emerges as a favorable choice for anesthesia in hemorrhoidectomy, demonstrating a lower incidence of urine retention and a prolonged analgesic effect in multiple hemorrhoidectomies. These findings support the conclusion that GAL represents an optimal anesthetic method for enhancing the postoperative experience in patients undergoing hemorrhoidectomy.

## 1 Introduction

Anal disorders are common problems that affect people's quality of life, and hemorrhoids are the most common anal disorder (1). Surgical treatment is the most effective modality for hemorrhoids (2). However, surgery is associated with discomfort, including severe pain and acute urinary retention (AUR) (3). The incidence of urine retention after a hemorrhoidectomy is 22%–37% (4, 5), mostly occurring within the first 24 h, and some patients subsequently require emergency medical attention.

Several studies have reported effective methods to reduce discomfort and complications after a hemorrhoidectomy, including changing the surgical method (6–8) and adjusting postoperative medications (painkillers and antibiotics) (9–12); however, studies on anesthesia modification are scarce (13). For more complicated anorectal surgeries, general anesthesia (GA) provides a stable depth of anesthesia to ensure the procedure is performed smoothly, and local infiltration blocks the nerves to prolong the analgesic effect. GA and local infiltration (GAL) can combine these advantages and produce multimodal analgesia. Spinal anesthesia (SA) blocking the spinal nerve may have a risk of urine retention and other discomfort (14). The incidence of AUR after hemorrhoidectomy under SA was 19.3% in a previous study (15). However, information on whether GA or SA is more suitable for ambulatory hemorrhoidectomy is poorly documented.

This study aimed to test the hypothesis that GAL results in a lower complication rate and better postoperative pain relief than SA alone after a hemorrhoidectomy.

## 2 Materials and methods

### 2.1 Study design and setting

This retrospective case-control study was approved by the Institutional Research Ethics Committee of Taichung Veterans General Hospital (IRB no. MD-340-2020) on June 14, 2022, and registered in ClinicalTrials.gov with the identifier NCT05571202 on October 7, 2022. The trial was conducted in compliance with the 2013 Helsinki Declaration. The study was designed as a retrospective research of medical records and was certified by the ethics committee as low-risk; hence, the requirement for consent was waived.

Data of patients who underwent hemorrhoidectomy from January 1, 2017 to March 31, 2023 were collected. All patients were hospitalized, and their data, including operation notes, nursing notes, pathology reports, discharge notes, and outpatient department medical records, were collected from the electronic health information system and electronic medical records. Patient data, including age, sex, American Society of Anesthesiologists (ASA) physical status score, anesthesia method, use of local infiltration or not, anesthesia duration, surgical position, hemorrhoidectomy number, use close or open methods, and postoperative analgesic drugs, were collected. Patients who used GA (including GA with an endotracheal tube, laryngeal mask, and intravenous GA) plus local infiltration (as described in the anesthesia and surgical methods) were classified into the experimental group (GAL group). Patients who used SA alone were included in the comparison group (SA group).

**Figure 1:**
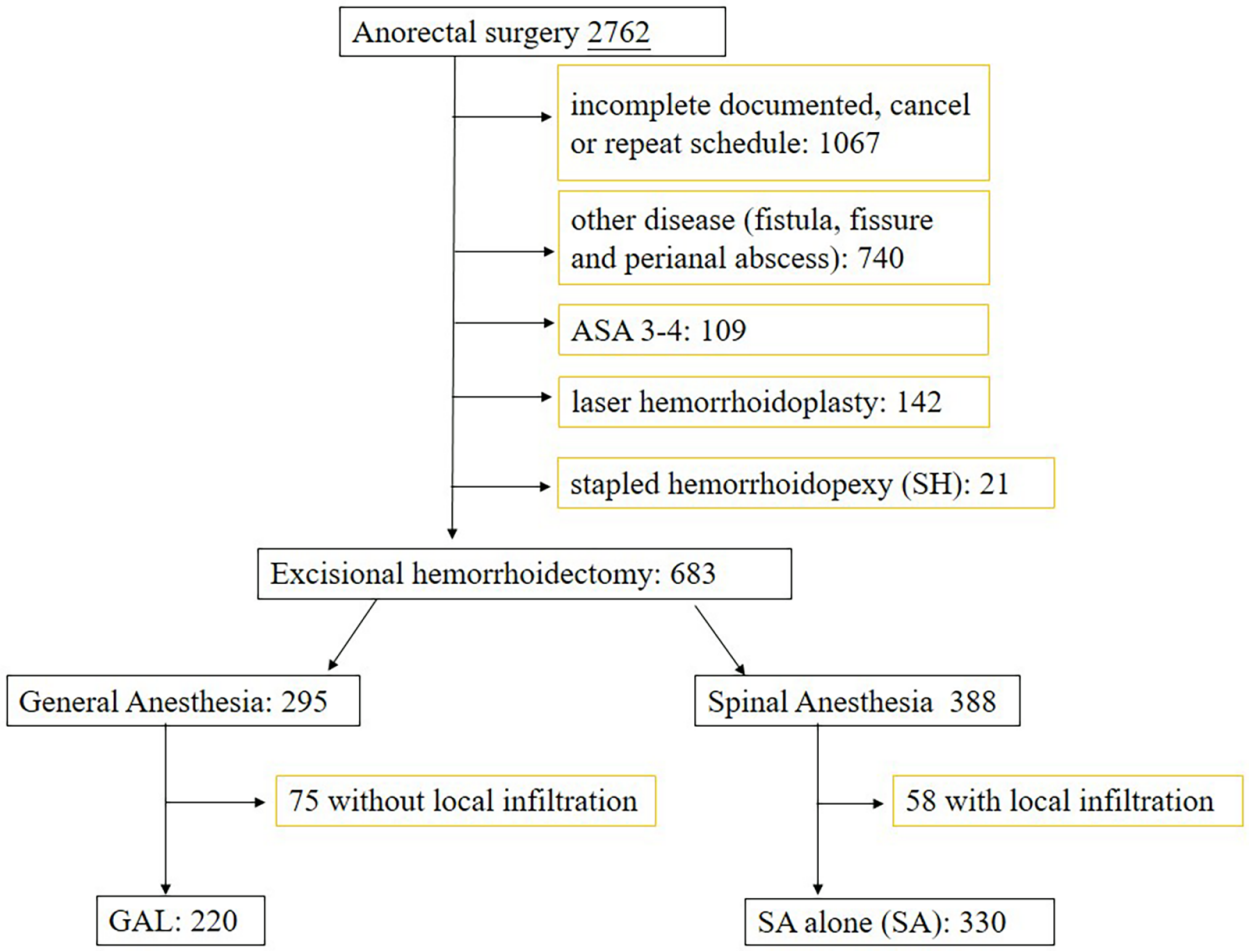
Schematic illustration of the study design. SH: stapled hemorrhoidopexy; GAL: general anesthesia with local infiltration; SA: spinal anesthesia alone; ASA: American Society of Anesthesiologist physical status.

The medical team acknowledged GAL, and informed consent for surgery was provided by each patient. The anesthetic method was decided based on the surgeon's discretion and the patient's preference. A flowchart of the study is presented in Figure 1.

### 2.2 Patient selection

The inclusion criteria were patients who underwent excisional hemorrhoidectomy. The principle of hemorrhoid surgery is to remove lesions with obvious symptoms, and the number of removals depends on the condition of the disease. The pain caused by the removal of multiple hemorrhoids will be more pronounced. For patients homogeneous between two anesthesia groups, the removal of one hemorrhoid is classified as a single hemorrhoidectomy, and the removal of two, three, or more hemorrhoids is classified as multiple hemorrhoidectomy. The exclusion criteria were patients who underwent other anorectal surgeries, including fistulectomy, fistulotomy, and fissurectomy. Patients who underwent other minimally invasive surgeries, such as ligation, hemorrhoidopexy, and laser hemorrhoidoplasty without hemorrhoid excision, patients <20 years old, and patients with an ASA risk score of ≥3 were also excluded.

### 2.3 Study size

To obtain sufficient sample power using the G*power software and Chi-square goodness-of-fit test, based on a similar study (5), we set GAL and SA urinary retention at 11% and 22%, the significance level of the test at 0.05, and the power to detect a difference at 0.9. The effect size indicating a medium effect was 0.35. Therefore, a total of 86 cases were needed in each group.

### 2.4 Anesthesia and surgical methods

All patients received peripheral intravenous fluid infusion with standard ASA monitors. Patients in the GA group received 0.1–0.2 mg glycopyrrolate, 1–2 μg/kg fentanyl, 1–2 mg/kg propofol, and 0.6–1.2 mg/kg cisatracurium. A laryngeal mask (the size depended on the patient's body weight: size 4 for weight 50–70 kg, size 5 for weight >70 kg) or endotracheal tube was inserted under the same anesthetic regimen, and an inhalation anesthetic was used for maintenance. Spontaneous respiration was maintained after the induction dose without cisatracurium for intravenous GA. A 26-gauge spinal needle was inserted at the L2–3 or L3–4 level for patients in the SA group. After the subarachnoid space was identified using the flow of the cerebrospinal fluid, 8 mg of 0.5% heavy bupivacaine (Marcaine Spinal Heavy; Astra Zeneca, Lund, Sweden) was injected. The patients were placed in a sitting position for 5 min, and the procedure was commenced.

**Figure 2:**
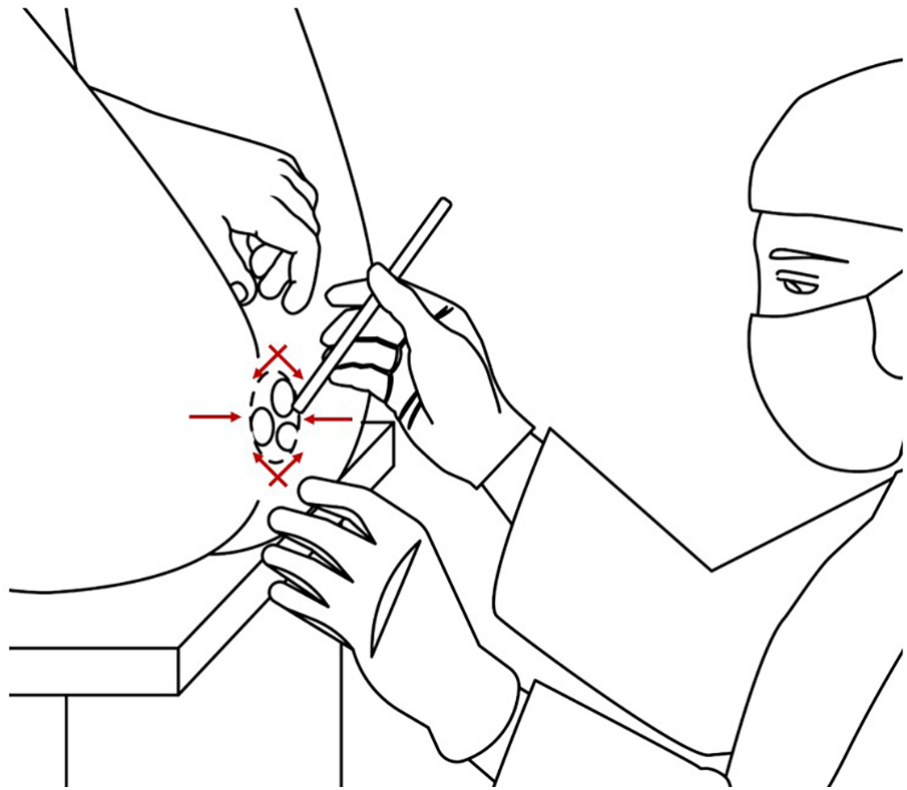
Lithotomy position during hemorrhoidectomy.

After the anesthetic procedure, the patient was placed in the jack-knife or lithotomy position. Most patients in the GA group were placed in the lithotomy position to protect their airways. The patient's legs were raised, similar to the position of a baby during a diaper change (Figure 2). For the patients in the jack-knife position, adhesive tape was used to separate the buttocks. The surgical field was well exposed for each position. The surgical field was prepared, and the procedure was performed using aseptic and antiseptic methods. For local infiltration, 40 ml of an anesthetic (20 ml normal saline + 20 ml 0.5% Bupivacaine (Marcaine® [5 mg/ml] or 20 ml 2% Xylocaine® [20 mg/ml] + 20 ml 0.5% Bupivacaine[Marcaine® (5 mg/ml)] was injected around the anus to achieve a nerve block effect. Hemorrhoids were exposed using a Ferguson retractor. After the symptomatic lesions were identified, the skin and mucosa were opened, and the hemorrhoid plexus was identified and detached from the internal sphincter muscle. The hemorrhoid pedicles were ligated using a simple tie and subjected to either electric coagulation or an energy device. In the open method, the hemorrhoid mucosa was left open; in the closed method, the mucosa was closed with chromic catgut or Vicryl sutures. Hemostasis was completed, and the site was compressed with an absorbable hemostatic gelatin sponge (Spongostan™; Johnson and Johnson Ethicon Inc., NJ, USA). The patients were transferred to the postoperative room and back to the ward after they became stable. Scheduled oral non-steroidal anti-inflammatory drugs and narcotic analgesics were administered postoperatively. Rescue analgesia (50 mg intravenous tramadol) was administered when the patients experienced intolerant pain [visual analog scale (VAS) ≥4, VAS 0 = no pain, VAS 10 = worst pain]. The patients were informed about possible complications and how to manage them, either in the ward or during home care. They were instructed to take a warm water sitz bath with clear water at least four times daily. One day after the operation, all records within 24 h of hospitalization were recorded in the electronic medical record. After the patients were discharged, all patients were followed up in our outpatient department at one week, three weeks, or four weeks postoperatively. The variables were recorded according to the outpatient medical records. If the patients returned to the emergency department due to complications, it would also be recorded in our system.

### 2.5 Study outcomes

The primary outcomes were the most frequent postoperative complications: pain, AUR, constipation, nausea, headache, and wound bleeding rate. These events were recorded according to CTCAE version 5.0 classifications (16). AUR was defined as grade 1 (feeling full of urine) and grade 2 (requiring catheterization). Constipation was defined as grade 1 (occasional symptoms) and grade 2 (persistent symptoms requiring laxatives). Bleeding was defined as grade 1 (mild symptoms without intervention) and grade 2 (symptoms requiring interventions). Nausea and headache were defined as documented in the medical record. Pain was recorded based on the VAS (0–10). Moderate pain was defined as VAS ≥4 at any point. The pain score at 0 h was recorded in the postoperative recovery room. The efficacy time of SA was 8 h, and the patients resumed ambulation and voiding. The VAS score after 24 h was recorded on the following day. AUR mostly occurred within 24 h; hence, the VAS scores at these three points were recorded. The secondary outcome was the pain score pattern.

### 2.6 Statistical analysis

Continuous variables are presented as medians and interquartile ranges regardless of the distribution. Categorical variables are represented as numbers and percentages. The Mann‒Whitney *U* and chi-square tests were used for between-group comparisons as appropriate. Factors associated with postoperative pain score and urinary retention were assessed by logistic regression in univariable analyses and multivariable models to calculate the adjusted odds ratio (OR). The effect of postoperative pain score was compared between the GAL and SA groups. *P *≤ 0.05 was considered statistically significant. All analyses were conducted using SPSS 22 (IBM Corp, New York, USA) and R version 4.1.3.

## 3 Results

In the center, 2,762 cases of anorectal surgeries were scheduled between January 1, 2017 and March 31, 2023. Of these, 1,067 cases were excluded due to incomplete records, no data on the procedure, and repeated cases, and 740 cases with other diseases, including fistula, fissure, and perianal abscess surgery, were excluded. Twenty-one cases of hemorrhoidopexy, 142 cases of laser hemorrhoidoplasty, and 109 cases with an ASA score of 3 were excluded. Finally, 533 excisional hemorrhoidectomy cases were included in the study. In this study, 295 cases used GA, and 388 cases used SA. We eliminated patients without local infiltration in the GA group and those with local infiltration in the SA group. Finally, there were 220 and 330 cases in the GAL and SA groups, respectively (Figure 1), with a median follow-up duration of 23.0 and 27.0 days. The proportion of multi-quadrant hemorrhoidectomy differed between the groups (GAL: 85.5% vs. SA: 79.6%), indicating a higher risk of pain and adverse effects. It would be necessary for the two groups to be homogeneous in terms of number of wounds (17). Therefore, cases of multiple hemorrhoidectomies were analyzed independently.

In the GAL group (*n* = 220), 165 (70.5%) of the patients used a laryngeal mask airway during anesthesia, 40 (26.8%) used heavy intravenous sedation, and 154 (70%) used the lithotomy position. The open method was more prevalent (71.4% vs. 52.7%, *P *< 0.001) and the anesthesia duration was longer (25.0 min vs. 20.0 min, *P *= 0.197) in the GAL group than in the SA group. The operation time was not significantly different in both groups. In the GAL group, 26.4% of the patients used the antiemetic drug granisetron. Postoperative analgesic medication use was not significantly different between the GAL and SA groups (Table 1).

**Table 1:** Demographic characteristics of patients that underwent single and multiple hemorrhoidectomies in the GAL and SA groups.

	Hemorrhoidectomy					Multiple hemorrhoidectomies				
	GAL (*n* = 220)		SA (*n* = 330)		*P*-value	GAL (*n* = 188)		SA (*n* = 261)		*P*-value
Age	51.0	(38.3–60.0)	49.0	(39.0–61.0)	0.790	52.0	(40–60)	49.0	(40.0–61.0)	0.690
Sex					0.622					0.478
Female	124	(56.4%)	193	(58.5%)		109	(58.0%)	160	(61.3%)	
Male	96	(43.6%)	137	(41.5%)		79	(42.0%)	101	(38.7%)	
BMI	23.7	(21.4–26.6)	23.8	(21.5–26.4)	0.677	23.7	(21.3–26.6)	23.6	(21.5–26.4)	0.835
ASA score					0.319					0.289
1	82	(37.3%)	137	(41.5%)		72	(38.3%)	113	(43.3%)	
2	138	(62.7%)	193	(58.5%)		116	(61.7%)	148	(56.7%)	
Anesthesia					<0.001**					<0.001**
GE	6	(2.7%)	0	(0.0%)		6	(3.2%)	0	(0.0%)	
GM	155	(70.5%)	0	(0.0%)		134	(71.3%)	0	(0.0%)	
IVG	59	(26.8%)	0	(0.0%)		48	(25.5%)	0	(0.0%)	
SA	0	(0.0%)	330	(100.0%)		0	(0.0%)	261	(100.0%)	
Local					<0.001**					<0.001**
No	0	(0.0%)	330	(100.0%)		0	(0.0%)	261	(100.0%)	
Yes	220	(100.0%)	0	(0.0%)		188	(100.0%)	0	(0.0%)	
Surgical position					<0.001**					<0.001**
Lithotomy	154	(70.0%)	9	(2.7%)		133	(70.7%)	8	(3.1%)	
Jack-knife	66	(30.0%)	321	(97.3%)		55	(29.3%)	253	(96.9%)	
Number of hemorrhoidectomies	2.0	(2.0–3.0)	2.0	(2.0–3.0)	0.023*	3.0	(2.0–3.0)	3.0	(2.0–3.0)	0.193
Hemorrhoidectomy number					0.079					–
One	32	(14.5%)	67	(20.4%)		0	(0.0%)	0	(0.0%)	
Multiple	188	(85.5%)	261	(79.6%)		188	(100.0%)	261	(100.0%)	
Methods					<0.001**					<0.001**
Open	157	(71.4%)	174	(52.7%)		136	(72.3%)	127	(48.7%)	
Close	63	(28.6%)	156	(47.3%)		52	(27.7%)	134	(51.3%)	
Emergency surgery	2	(0.9%)	9	(2.7%)	0.214	2	(1.1%)	7	(2.7%)	0.315
Anesthesia time	25.0	(18.0–30.0)	20.0	(15.0–30.0)	0.197	25.0	(17.0–30.0)	20.0	(15.030.0)	0.512
OP time, m	29.0	(19.0–37.5	24.0	(17.0–39.0)	0.175	29.0	(20.0–39.0)	29.0	(19.5–40.0)	0.555
Intraoperative fluid	500.0	(400.0–500.0)	500.0	(300.0–500.0)	0.054	500.0	(400.0–500.0)	500.0	(300.0–500.0)	0.224
Regular post-op analgesics					0.743					0.813
Nil	9	(4.1%)	13	(3.9%)		8	(4.3%)	11	(4.2%)	
Single	110	(50.0%)	176	(53.3%)		93	(49.5%)	137	(52.5%)	
Double	101	(45.9%)	141	(42.7%)		87	(46.3%)	113	(43.3%)	
Opioids	138	(62.7%)	228	(69.1%)	0.121	119	(63.3%)	181	(69.3%)	0.179
NSAIDs	174	(79.1%)	230	(69.7%)	0.015*	148	(78.7%)	182	(69.7%)	0.033*
Rescue pain control	105	(47.7%)	180	(54.5%)	0.117	94	(50.0%)	153	(58.6%)	0.070
Kytron	58	(26.4%)	3	(0.9%)	<0.001**	52	(27.7%)	3	(1.1%)	<0.001**
Urine retention	35	(15.9%)	102	(30.9%)	<0.001**	33	(17.6%)	89	(34.1%)	<0.001**
Follow-up time, day	23.0	(18.0–40.0)	27.0	(14.0–40.0)	0.179	24.0	(19.0–40.0)	29.0	(15.0–43.0)	0.065

GAL, general anesthesia with local infiltration; SA, spinal anesthesia alone; GE, general anesthesia with an endotracheal tube; GM, general anesthesia through laryngeal mask; IVG, intravenous general anesthesia; OP, operative; NSAIDs, non-steroidal anti-inflammatory drugs; BMI, body mass index; ASA, American Society of Anesthesiologists.

Chi-square test or Mann-Whitney U test, median (IQR); *P<0.05, **P<0.01

The proportion of patients with moderate pain (VAS ≥4) was similar between the GAL and SA groups (36.2% vs. 39.8%, *P *= 0.429), but rescue analgesia was less frequent in multiple hemorrhoidectomy cases (50% vs. 58%, *P *= 0.070). AUR was significantly lower in the GAL group than in the SA group (15.5% vs. 32.1%, *P *< 0.001). Other surgical complications, including constipation and wound bleeding, were similar between both groups. The overall proportion of adverse effects was lower for multiple hemorrhoidectomies in the GAL group compared to that in the SA group (56.9% vs. 67.4%, *P *= 0.023). The occurrences of nausea and headache were similar in both groups. The occurrence of wound pain in the first week was similar in both groups (83.5% vs. 79.3%, *P *= 0.262) (Table 2).

**Table 2:** Outcomes of hemorrhoidectomy in the GAL and SA groups.

	Hemorrhoidectomy					Multiple hemorrhoidectomies				
	GAL (*n* = 220)		SA (*n* = 330)		*P*-value	GAL (*n* = 188)		SA (*n* = 261)		*P*-value
Pain symptoms	20	(9.1%)	0	(0.0%)	<0.001**	19	(10.1%)	0	(0.0%)	<0.001**
Pain grade					<0.001**					<0.001**
0	28	(12.7%)	87	(26.4%)		20	(10.6%)	54	(20.7%)	
1	172	(78.2%)	243	(73.6%)		149	(79.3%)	207	(79.3%)	
2	20	(9.1%)	0	(0.0%)		19	(10.1%)	0	(0.0%)	
AUR	34	(15.5%)	106	(32.1%)	<0.001**	33	(17.6%)	93	(35.6%)	<0.001**
AUR grade					<0.001**					<0.001**
0	186	(84.5%)	224	(67.9%)		155	(82.4%)	168	(64.4%)	
1	2	(0.9%)	0	(0.0%)		2	(1.1%)	0	(0.0%)	
2	32	(14.5%)	106	(32.1%)		31	(16.5%)	93	(35.6%)	
Constipation	14	(6.4%)	19	(5.8%)	0.769	12	(6.4%)	18	(6.9%)	0.830
Constipation grade				0.718					0.789	
0	206	(93.6%)	311	(94.2%)		176	(93.6%)	243	(93.1%)	
1	1	(0.5%)	3	(0.9%)		1	(0.5%)	3	(1.1%)	
2	13	(5.9%)	16	(4.8%)		11	(5.9%)	15	(5.7%)	
Nausea					0.909					0.543
0	215	(97.7%)	322	(97.6%)		184	(97.9%)	253	(96.9%)	
1	5	(2.3%)	8	(2.4%)		4	(2.1%)	8	(3.1%)	
Headache	0	(0.0%)	3	(0.9%)	0.279	0	(0.0%)	3	(1.1%)	0.268
Wound bleeding					0.044*					0.167
0	197	(89.5%)	313	(94.8%)		169	(89.9%)	246	(94.3%)	
1	21	(9.5%)	14	(4.2%)		17	(9.0%)	12	(4.6%)	
2	2	(0.9%)	3	(0.9%)		2	(1.1%)	3	(1.1%)	
Any of the symptoms above				0.317					0.098	
0	140	(63.6%)	196	(59.4%)		117	(62.2%)	142	(54.4%)	
1	80	(36.4%)	134	(40.6%)		71	(37.8%)	119	(45.6%)	
ED readmission	2	(0.9%)	4	(1.2%)	1.000	2	(1.1%)	4	(1.5%)	1.000

The six most frequent complications after hemorrhoidectomy were pain (VAS ≥4) in 24 h, AUR, constipation, nausea, headache, and bleeding. The ED readmission rate was defined as symptomatic pain within 7 days. AUR was defined as grade 1 (feeling full of urine) and grade 2 (requiring catheterization). Constipation was defined as grade 1 (occasional symptoms) and grade 2 (persistent symptoms requiring laxatives). Bleeding was defined as grade 1 (mild symptoms without intervention) and grade 2 (symptoms requiring interventions).

Chi-square test or Mann-Whitney U test, median (IQR). *P<0.05, **P<0.01. AUR: acute urinary retention

**Figure 3:**
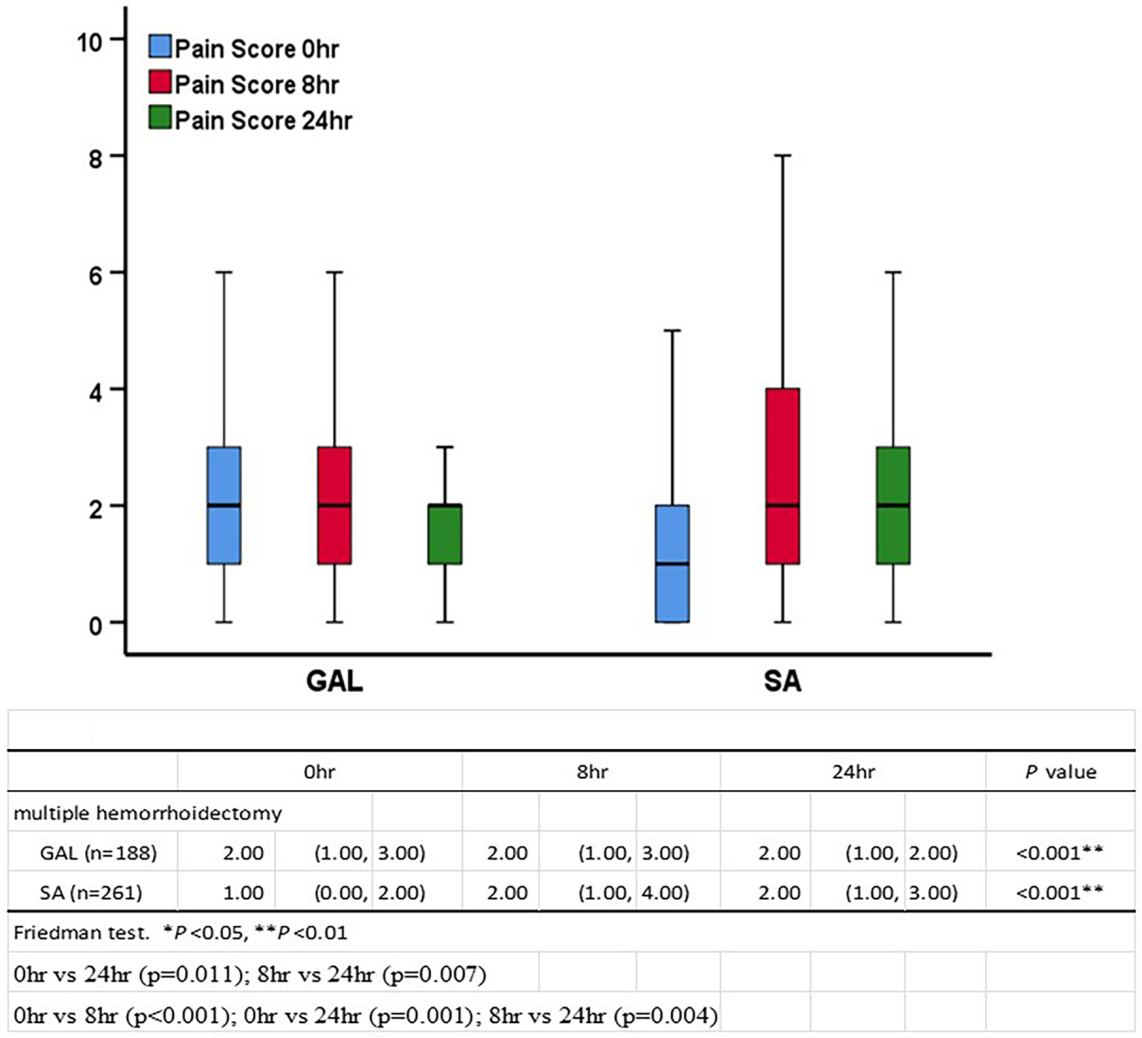
VAS at 0, 8, and 24 h between the GAL and SA groups in multiple hemorrhoidectomies. Comparison of the VAS pain score between the two groups. VAS: visual analog scale, an 11-point scale where 0 = no pain and 10 = worst pain.

Patients in the SA group had a significantly higher pain score at 8 h than at 0 h (2.00 vs. 1.00, *P *< 0.001), which then decreased at 24 h (2.00 vs. 2.00, *P *= 0.001). Patients in the GAL group had a relatively consistent pain score at 0, 8, and 24 h (Figure 3).

In the multivariate analysis, the risk factors for AUR were age >60 years old [OR: 1.02 (1.01–1.04), *P *= 0.003], multiple hemorrhoidectomy [OR: 2.30 (1.22–4.31, *P *= 0.010)], rescue analgesia [OR: 1.76 (1.16–2.68), *P *= 0.008], and SA alone [OR: 2.44 (1.57–3.81), *P *< 0.001] (Table 3).

**Table 3:** Risk factor for acute urine retention.

	0 h		8 h		24 h			*P-*value
Hemorrhoidectomy								
GAL (*n* = 220)	2.00	(1.00, 3.00)	2.00	(1.00, 3.00)	2.00	(1.00, 2.00)	<0.001**	
SA (*n* = 330)	1.00	(0.00, 2.00)	2.00	(1.00, 4.00)	2.00	(1.00, 3.00)	<0.001**	<0.001**
Multiple hemorrhoidectomies								
GAL (*n* = 188)	2.00	(1.00, 3.00)	2.00	(1.00, 3.00)	2.00	(1.00, 2.00)	<0.001**	<0.001**
SA (*n* = 261)	1.00	(0.00, 2.00)	2.00	(1.00, 4.00)	2.00	(1.00, 3.00)	<0.001**	<0.001**

Friedman test; GAL, general anesthesia plus local infiltration; SA, spinal anesthesia.

*Post hoc* analysis (Bonferroni) revealed *P *< 0.05; 0 h vs. 24 h (*P *= 0.002); 8 h vs. 24 h (*P *= 0.010); 0 h vs. 8 h (*P *< 0.001); 0 h vs. 24 h (*P *< 0.001); 8 h vs. 24 h (*P *= 0.001); 0 h vs. 24 h (*P *= 0.011); 8 h vs. 24 h (*P *= 0.007); 0 h vs. 8 h (*P *< 0.001); 0 h vs. 24 h (*P *= 0.001); 8 h vs. 24 h (*P *= 0.004).

*P < 0.05. **P < 0.01.

## 4 Discussion

The current study found that GAL lowers the urine retention rate and extends the analgesic effect in multiple hemorrhoidectomies. There was no difference in the occurrence of other complications and the readmission rate.

Many minimally invasive treatments, including ligation, stapled hemorrhoidopexy (7), and laser hemorrhoidoplasty (6), are available for hemorrhoids; however, they all have limitations. Complicated cases, such as fourth-degree, mixed-type, and rosette-type hemorrhoids with thrombosis, require excision surgery (2, 18). Pain and AUR are the most common acute complications that require medical attention after anal surgery (3). The inability to relax the pelvic floor sphincter due to pain affects the coordinated operation of the genitourinary system (12). Hemorrhoidectomy and fistulectomy have different levels of AUR risk (5.8% vs. 19.4% in the external cohort data of this study and previous studies) (4), probably because the levels of the initial pain differ. Patients with perianal abscess or anal fistula initially experienced pain; after the procedure, they felt relief even if there was a wound. Therefore, the risk of AUR was very low. In a previous study, older age, a high number of resected hemorrhoids, and the use of supplementary analgesic drugs were independent risk factors for AUR (14), similar to the present study.

The most common symptoms of patients with hemorrhoids before surgery were bleeding and prolapse without pain. The anus is very sensitive, and the postoperative wound pain makes the patients uncomfortable, leading to a high AUR risk. Pain also causes a substantial reduction in satisfaction with a hemorrhoidectomy.

Many studies have been conducted on pain reduction after anal surgery; overall, a multimodal pain control approach is required (19). This approach is generally divided into several parts: (a) preoperative, which involves colon cleansing before surgery and avoiding spicy foods to reduce fecal contamination and stimulation of the wound, and (b) perioperative, in which enough skin and mucosal membranes should be preserved, and only symptomatic lesions should be removed; one should avoid removing too much tissue. Total hemorrhoidectomy has a high complication risk and pain score.

The energy device LigaSure^TM^ (Metronic Inc., MD, USA) or a Harmonic scalpel is used to increase the accuracy of the procedure and hemostasis (8, 20). The impact of open or closed methods on wound pain is unknown (21, 22). A meta-analysis reported that the closed method had better results (23). However, a randomized controlled trial reported a similar pain index for the open and closed methods (24). In the present study, the open method did not increase pain within 24 h. After the procedure, the wound is kept clean, and the patient takes a warm water sitz bath as soon as possible and is administered oral or injection analgesics. However, the side effects of non-steroidal anti-inflammatory drugs, including nephrotoxicity, gastric ulcer, nausea, vomiting, dizziness, and allergic reactions, limit their usage and effectiveness. Other atypical pain relief methods, such as botulinum toxin injection (2), moxibustion (25), and oral flavonoids (11), have been documented but are not as popular and effective. Although the oral antibiotic metronidazole can reduce pain (9, 10), it has a risk of gastrointestinal side effects.

In the past, only a few studies investigated the anesthetic methods for anal surgery (13, 26, 27). A study used the saddle block technique in outpatient anal surgery, with an acceptable AUR risk (1.7%) (28, 29); however, the popularity of this technique limits its application. A study comparing three types of anesthesia (GA, SA, local) found that local anesthesia reduced AUR without the side effects of GA and SA (13). However, the study's sample size was too small, and the differences between the patients in each group were large; records on the effectiveness of the depth of anesthesia during the procedure were also lacking. In clinical practice, inadequate anesthesia depth occurs with pure local infiltration, which affects the success of the procedure and may lead to local anesthesia systemic toxicity (30). With recent advancements in anesthesia technology and drugs, the occurrence of nausea and vomiting has been greatly reduced. In the present study, 26% of the patients in the GAL group were treated with a self-paid 5-HT3 antagonist (granisetron), and 2.3% of the patients experienced postoperative nausea and vomiting, similar to the proportion in the SA group. For patients using GAL, the lying down lithotomy posture is more reliable and safer for airway and cardiopulmonary function compared to the prone posture in patients using SA (31). Surgeons can perform hemorrhoidectomy in a stable state with a good field of view, which improves the quality of the procedure.

Patients using SA require bed rest for 6–8 h, which may affect urination. The risk of AUR is high with excess intravenous fluid administration. Patients using GAL can achieve ambulation early to urinate. Several methods exist to reduce postoperative pain, including teaching patients to clean the wound postoperatively, proper nutrition to facilitate wound healing, and quitting smoking. Oral painkillers can greatly reduce the discomfort of a hemorrhoidectomy. GAL is especially suitable for complex anal surgery, especially in multiple hemorrhoidectomies.

Our study had some limitations. First, although the follow-up time in this study was 26 days, the primary outcome observation time was very short, and only the pain score and AUR events within 24 h were recorded. More objective evidence can be provided if there are additional records of pain scores and patient satisfaction scores. Second, some confounding factors exist between the experimental and control groups, including different surgeons, different surgical methods, and inconsistent use of postoperative painkillers. Third, half of the patients were excluded for incomplete data in this retrospective setting. Further prospective studies that include integrated multi-model analgesia regimens are needed to overcome these limitations.

In conclusion, GAL lowers the urine retention rate and produces a more stable analgesic effect in multiple hemorrhoidectomies than SA. Therefore, GAL is an optimal anesthesia method for hemorrhoidectomy, as it reduces pain and urinary retention in patients and is more suitable for outpatient surgery.

## Data Availability

The raw data supporting the conclusions of this article will be made available by the authors, without undue reservation.
